# Machine Learning of Single Cell Transcriptomic Data From anti-PD-1 Responders and Non-responders Reveals Distinct Resistance Mechanisms in Skin Cancers and PDAC

**DOI:** 10.3389/fgene.2021.806457

**Published:** 2022-02-01

**Authors:** Ryan Liu, Emmanuel Dollinger, Qing Nie

**Affiliations:** ^1^ Department of Mathematics, University of California, Irvine, Irvine, CA, United States; ^2^ Department of Developmental and Cell Biology, University of California, Irvine, Irvine, CA, United States; ^3^ Center for Complex Biological Systems, University of California, Irvine, Irvine, CA, United States; ^4^ NSF-Simons Center for Multiscale Cell Fate Research, University of California, Irvine, Irvine, CA, United States

**Keywords:** immunotherapy, machine learning of single cell sequencing, therapeutic response prediction, supervised learning, deep learning, single-cell transcriptomic sequencing, basal cell carcinoma, pancreatic ductal adenocarcinoma

## Abstract

Immune checkpoint therapies such as PD-1 blockade have vastly improved the treatment of numerous cancers, including basal cell carcinoma (BCC). However, patients afflicted with pancreatic ductal carcinoma (PDAC), one of the deadliest malignancies, overwhelmingly exhibit negative responses to checkpoint therapy. We sought to combine data analysis and machine learning to differentiate the putative mechanisms of BCC and PDAC non-response. We discover that increased MHC-I expression in malignant cells and suppression of MHC and PD-1/PD-L expression in CD8^+^ T cells is associated with nonresponse to treatment. Furthermore, we leverage machine learning to predict response to PD-1 blockade on a cellular level. We confirm divergent resistance mechanisms between BCC, PDAC, and melanoma and highlight the potential for rapid and affordable testing of gene expression in BCC patients to accurately predict response to checkpoint therapies. Our findings present an optimistic outlook for the use of quantitative cross-cancer analyses in characterizing immune responses and predicting immunotherapy outcomes.

## 1 Introduction

Cancer immunotherapy has shown to elicit substantial response to many cancers and has led to significant increases in quality of life for cancer patients. This is especially true of checkpoint therapy, which causes tumor regression in previously untreatable cancers. Response to checkpoint therapy has been positively correlated with tumor mutational burden (TMB) and with presence of CD8^+^ T cells in the tumor microenvironment (characterized as “hot” tumors) (Yarchoan et al., 2017) (Tumeh et al., 2014). However, the potential mechanisms of checkpoint therapy are still being investigated and there are as of yet few prognostic markers for response (Bai et al., 2020). Potential biomarkers include the alteration of signaling pathways in tumor cells, namely mutations in the interferon (IFN)-*γ* pathway, as well as pathways related to tumor cell proliferation and infiltration (Possick, 2017). Poor response to immunotherapy is also linked to inactivation of *PTEN*, mutations of *POLE*, and linked mutations in *KRAS* and *STK11* (Wang et al., 2021).

Basal cell carcinoma (BCC) is a skin cancer with high TMB (estimates range from a median of 47.3 mutations/Mb to 65 mutations/Mb), arising from skin membrane stem cells (Chalmers et al., 2017) (Bonilla et al., 2016). Despite being mostly characterized as a “cold” tumor, BCC has been shown to exhibit partial and complete responses to checkpoint therapy (Moujaess et al., 2021) (Walter et al., 2010). Recently, the PD-1 inhibitor cemiplimab received FDA approval for patients with advanced non-resectable BCC that are resistant to Hedgehog pathway inhibition (Moujaess et al., 2021). BCC is a relatively unique cancer in that the TMB does not correlated with immunogenicity. This is thought to be a combination of downregulation of major histocompatability complex class I (MHC-I) expression and immunosuppression via influx of T regulatory cells driven by overexpression of the Hedgehog pathway (Walter et al., 2010) (Grund-Gröschke et al., 2020).

Pancreatic ductal adenocarcinoma, a carcinoma arising from ductal cells in the pancreas, is in essence an incurable disease, with less than 5% survival rate over 5 years as of 2016 (Bengtsson et al., 2020). This survival rate, in conjunction with projections that pancreatic cancers will be one of the major causes of cancer-related deaths by 2030, highlights a strong need to develop better biomarkers and treatments (Rahib et al., 2014). Despite significant progress having been made in oncology treatment, PDAC has proven to be incredibly challenging to treat, due to a multitude of factors including lack of symptoms before metastasis and lack of specific clinical characteristics (Wolfgang et al., 2013). PDAC has also been found to be non-responsive to checkpoint immunotherapy, showing a poor response to CTLA-4, PD-1 and PD-L1 therapies (Royal et al., 2010) (Renouf et al., 2020). The reasons for this lack of response are still under study; proposed factors include levels of microsatellite instability, tumor infiltrating lymphocytes (TILs), and DNA mismatch repair deficiency (Christenson et al., 2020) (Pu et al., 2019). Although it has a relatively low TMB, PDAC has a highly immunosuppressive tumor microenvironment and is immunogenic (Fan et al., 2020).

**FIGURE 1:**
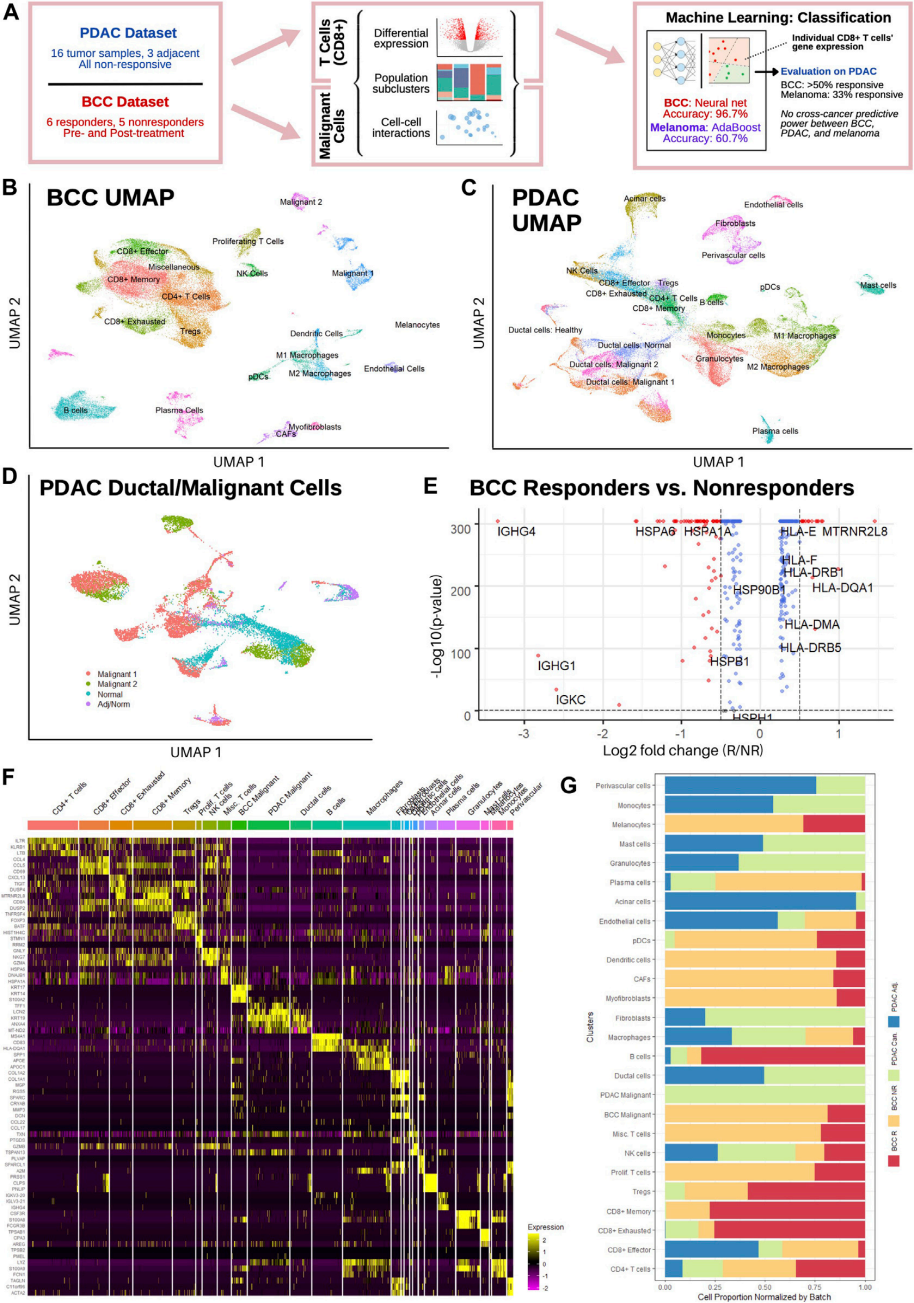
Single-cell sequencing reveals distinct T cell subpopulations in BCC and PDAC and ductal cell subpopulations in PDAC. **(A)** Workflow diagram. **(B,C)** Dimensional reduction of **(B)** BCC and **(C)** PDAC TME. **(D)** Dimensional reduction of PDAC ductal cells. **(E)** Differential gene expression between BCC responders and nonresponders; positive fold change indicates greater expression in responders. **(F)** Single-cell resolution heatmap of top three differentially expressed genes per cluster in merged BCC and PDAC dataset. **(G)** Normalized proportion of cells in each cluster identified in **(F)** that belong to BCC responders (BCC R), BCC nonresponders (BCC NR), PDAC tumors (PDAC Can.), and adjacent PDAC samples (PDAC Adj.).

In order to study the differential mechanisms by which BCC and PDAC cancers resist checkpoint immunotherapy treatment and building on our previous work (Dollinger et al., 2020), we leveraged two recent single-cell transcriptomic datasets of PDAC and BCC (Figures 1A and Supplementary Figure S1). Through comparing these two datasets, we identified potential common biomarkers for nonresponse to PD-1 blockade and differences in the immune mechanisms combating tumor progression in these two cancers. We found that PDAC suppresses MHC-I gene expression in CD8^+^ T cells and upregulates MHC-I in malignant cells compared to BCC. Furthermore, the PD-1/PD-L signaling axis is significantly weaker in PDAC, leaving diminished opportunity for phenotypic changes to occur through boosting its activity. Utilizing machine learning classification algorithms, we additionally discovered that PDAC displays greater similarities to melanoma, which is highly immunogenic and undergoes rapid metastasis, than to BCC (Dollinger et al., 2020).

## 2 Results

### 2.1 Characterization of the BCC and PDAC TME

In order to characterize the transcriptomic differences between responders and non-responders to PD-1 blockade therapy, we analyzed a previously published scRNA-seq dataset of basal cell carcinoma patients pre- and post-treatment (Yost et al., 2019). The dataset consists of 24 site-matched samples from 11 patients with advanced BCC; a total of 53,030 malignant, immune, and stromal cells were obtained between the six responsive and five nonresponsive patients. Unsupervised clustering of the dataset revealed 20 distinct clusters (Figure 1B), including 8 T cell clusters and two malignant cell clusters (Methods). Our clustering largely agrees with the original analysis (Supplementary Figure S2A), with the exceptions that we only found 1 B cell cluster and differentiated macrophages into the M1/M2 polarization as defined in (Orecchioni et al., 2019).

Separately, a dataset of 46,244 cells from 16 PDAC patients and 8,541 cells from three non-malignant adjacent samples was used to characterize the PDAC TME (Steele et al., 2020); all samples were taken before any treatment and include both surgical and fine-needle biopsy specimens. Both the malignant and adjacent samples were integrated together before clustering, which revealed 22 distinct subpopulations (Figure 1C). Whereas the general cluster labels correspond with those of the original paper, two important distinctions are made. First, CD8^+^ T cells are divided into effector/activated cells, memory cells, and chronically activated/exhausted cells, referred to hereafter as exhausted cells; these labels correspond with the CD8^+^ T cell subclusters in the BCC dataset to facilitate further direct comparison, and are therefore not equivalent to those in Extended Data Figure 4 of the original analysis. However, examination of mean scaled expression of highly enriched genes reveals that the newly defined clusters are transcriptomically similar to those in the original analysis (Supplementary Figure S2B). Second, within the population of epithelial/ductal cells, two distinct clusters of malignant cells were identified using 205 marker genes commonly upregulated in PDAC tumor samples (Figure 1D) (Tang et al., 2018). The identification of these clusters is novel and was not detected by the original authors. Whereas one malignant cluster had significantly elevated expression of nearly all marker genes and a high percentage (>50%) of all cells expressing each gene, the second malignant cluster had much more sparse and less significantly elevated expression of the DEGs, suggesting that there exists a wide spectrum in the degree of malignancy of ductal cells (Supplementary Figure S2C). Both normal ductal cells in malignant PDAC samples and those from adjacent samples exhibited negligible expression of the 205 marker genes.

Comparing average gene expression across all cells between responders and nonresponders in BCC, we found that MHC genes are overexpressed in responders, whereas heat shock protein (*HSP*) genes are overexpressed in nonresponders (Figure 1E). This is in line with current literature: reduced MHC-I expression is well-known to facilitate immune evasion (Šmahel, 2017); MHC-II expression is correlated with response to PD-1 blockade treatment (Rodig et al., 2018); and *HSP* genes are associated with tumor proliferation and metastasis (Ciocca and Calderwood, 2005). Merging both datasets, we find that the top differentially expressed genes (DEGs) for each cluster aligns with the marker genes used to identify them in (Yost et al., 2019) and (Steele et al., 2020) (Figure 1F, Methods). Furthermore, no significant difference was detected in the expression of top DEGs in each cluster, e.g. expression of *CCL4*, *CCL5*, and *CD59* is similar between PDAC and BCC CD8^+^ effector T cells. However, wide discrepancies can be seen in the relative populations of different clusters between BCC responders, BCC nonresponders, and PDAC patients (Figure 1G). We find that BCC responders are more heavily represented amongst B cells and T cells, whereas BCC nonresponders have greater numbers of stromal, myeloid, and malignant cells, recapitulating previous analyses (Dollinger et al., 2020) (Yost et al., 2019). Meanwhile, PDAC tumors have very low numbers of B cells and T cells in comparison to all BCC tumors, but have much larger populations of macrophages and endothelial cells. This highlights the challenges of using immunotherapy in PDAC; it also justifies the comparison of two different cancers due to the similarities in cell population between non-responders in BCC and PDAC.

### 2.2 CD8+ T Cells Are More Active in BCC Than PDAC

One of the main functions of PD-1 blockade is to reinvigorate exhausted CD8^+^ T cells, leading to a stronger anti-tumor response and eventual tumor regression (Verma et al., 2019); thus, the altered function and composition of T cells is a primary suspect in the nonresponse of PDAC to immunotherapies. Due to the absence of data on PDAC responders, in this section we compare T cells in PDAC to those in BCC responders and nonresponders. Similarities in composition or gene expression between the T cells of PDAC and BCC nonresponders, as well as commonalities in the differences between BCC responders and nonresponders and the differences between BCC responders and PDAC, provide potential factors for further study.

**FIGURE 2:**
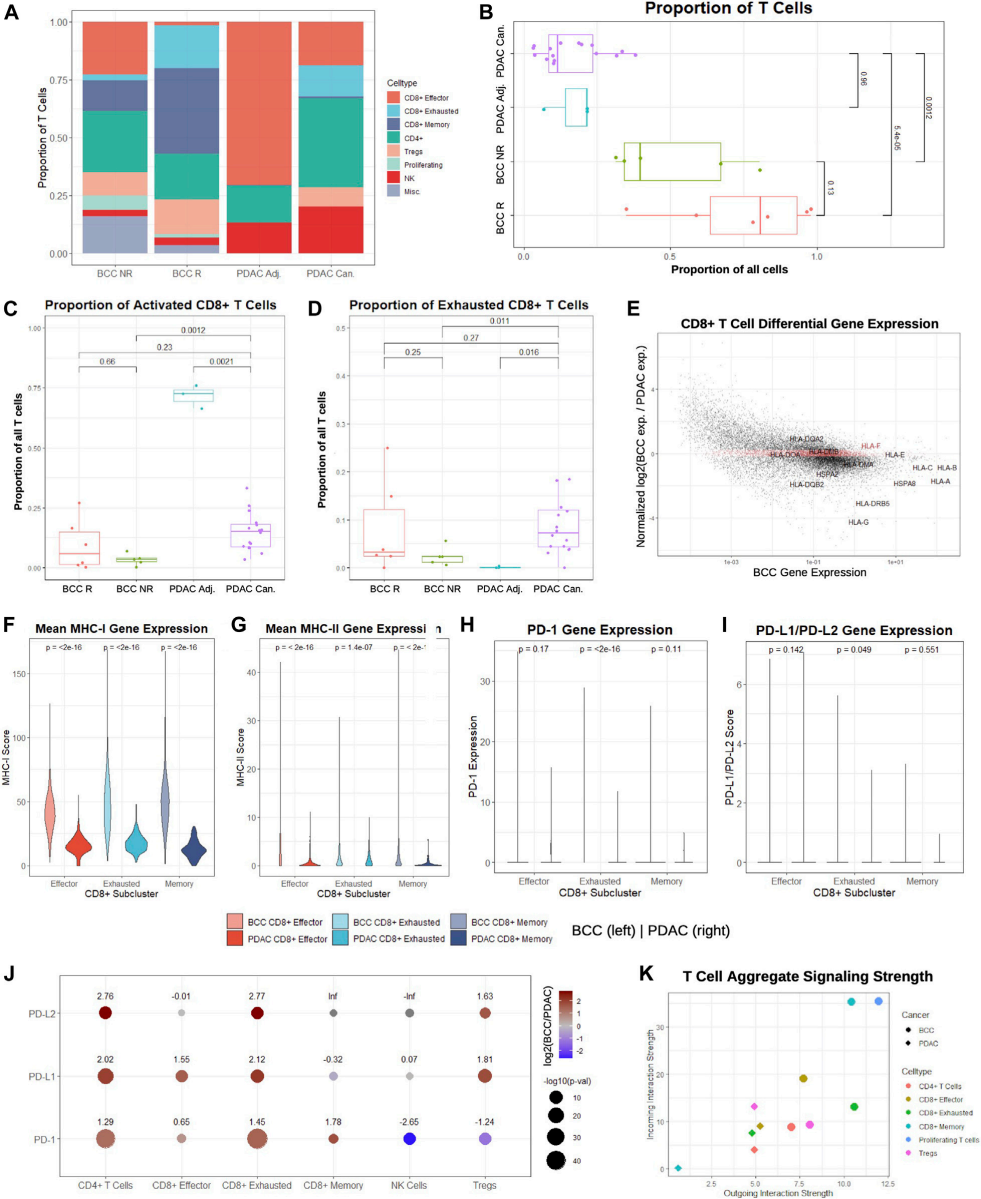
CD8^+^ T cells in BCC exhibit higher signaling strength and MHC-I/PD-1 pathway expression compared to PDAC. **(A)** Proportion of T cell subclusters in BCC (R = Responsive, NR = Nonresponsive) and PDAC (Can. = Cancerous, Adj. = Adjacent) samples. **(B)** Per-patient comparison of the proportion of all cells that are T cells. **(C, D)** Per-patient comparison of the proportion of T cells that are **(C)** CD8^+^ effector and **(D)** CD8^+^ exhausted. **(E)** Differential gene expression in CD8^+^ T cells, BCC vs PDAC (positive fold change indicates higher expression in BCC); the top 2000 genes with the lowest absolute value normalized fold change in expression are highlighted in red. **(F-I)** Per-cell comparison between BCC and PDAC CD8^+^ T cell subclusters on **(F)** mean MHC-I, **(G)** mean MHC-II, **(H)** PD-1, and **(I)** mean PD-L1/PD-L2 gene expression. **(J)** Comparison of fold change between average PD-1 pathway gene expression of BCC and PDAC T cell subclusters; positive values indicate greater expression in BCC. **(K)** Comparison of aggregate outgoing and incoming signaling strength between BCC and PDAC T cell subclusters.

Comparing the T cell populations in PDAC tumor sites and adjacent samples, we find significant differences in relative subpopulation sizes – in particular, there are virtually no regulatory T cells (Tregs), memory CD8^+^ T cells, or exhausted CD8^+^ T cells in adjacent samples (Figure 2A). This suggests that a subset of effector CD8^+^ T cells in the PDAC TME enter an exhausted phenotype or differentiate into memory cells after prolonged exposure to cancer (Xia et al., 2019). Furthermore, we unexpectedly find a substantially larger population of CD8^+^ exhausted and memory cells in BCC responders and a diminished number of CD8^+^ effector cells. Therefore, it is possible that BCC responders benefit more from PD-1 therapy due to greater potential for phenotypic shifts on the cellular level, or simply that T cells in responders have experienced prolonged exposure to the malignancy. Both PDAC populations lack proliferating T cells, supporting its reputation as extremely immunosuppressive (Foucher et al., 2018).

To determine whether these trends are patient-specific, we first compared the fraction of all cells in each pre-treatment sample that are identified as T cells (Figure 2B). As expected, pre-treatment responders have a greater proportion of T cells than nonresponders, although the difference is statistically insignificant (*p* > 0.05). However, both BCC responders and nonresponders have significantly higher T cell proportions than both malignant and adjacent PDAC samples by a factor of 4–8. Comparing the proportion of T cells classified as activated and exhausted, we find that the proportions are similar between all patients, with the unexpected exception that the vast majority of T cells in the adjacent pancreas samples are effector CD8^+^ T cells (Figures 2C, D). This may indicate that adjacent samples may not reflect a true negative control, as is often used in the literature.

Identification of the top 2,000 genes with the most similar gene expression between BCC and PDAC unsurprisingly reveal no notable gene groups, supporting the theory that the two cancers rely on different systems of immune activation. However, we notice that *HLA* genes are amongst the most highly enriched genes in both cancers; furthermore, they are consistently overexpressed in BCC compared to PDAC by a factor of 2-10 with the exception of *HLA-E* and *HLA-F*, suggesting that PDAC suffers from much more severe MHC-I suppression (Figure 2E). To confirm whether these differences hold on a patient level, we constructed a MHC-I and MHC-II score (Methods). Comparison of the per-patient MHC-I scores between BCC and PDAC for each of the CD8^+^ T cell subclusters shows that regardless of the subcluster, BCC CD8^+^ T cells have significantly elevated MHC-I expression in comparison to PDAC; this discrepancy is most pronounced in memory CD8^+^ T cells, where MHC-I scores are on average 4 times higher (Figure 2F). Similarly, per-patient comparison of MHC-II scores show that all three groups of CD8^+^ T cells have significantly lower expression in PDAC than BCC - in particular, the majority of effector and memory CD8^+^ T cells in PDAC exhibit virtually no MHC-II expression (Figure 2G). This supports prior research demonstrating that MHC-I molecules are degraded by autophagy-dependent mechanisms in PDAC, thereby facilitating impaired antigen presentation and resistance to checkpoint therapies (Johnson et al., 2016); no such mechanisms have been implicated in BCC and this provides evidence against such a mechanism existing in the BCC TME.

We then compared the distribution of PD-1 and PD-L1/PD-L2 expression in BCC and PDAC CD8^+^ T cells (Figures 2H, I). In both cancers, the vast majority (>95%) of cells exhibit zero expression in all subclusters; the only exception is exhausted CD8^+^ T cells in BCC. Thus, no significant difference (*p* > 0.01) is detected between BCC and PDAC in the expression distribution of the PD-1 pathway. Due to the exceptionally low expression in all clusters, we examined the mean expression of all cells in each T cell subcluster (Figure 2J). We find that with the exception of NK cells and Tregs in the expression of PD-1, BCC T cells are also upregulated in PD-1/PD-L1/PD-L2 in all subclusters by a factor of 3–7. These results imply that a combination of immune suppression and low expression of the MHC-I and PD-1 gene pathway in CD8^+^ T cells contribute to decreased response to PD-1 blockade in PDAC, as there is not sufficient expression of the PD-1 pathway to induce significant change in T cell activity with PD-1 blockade.

Lastly, we compared the aggregate signaling strength of each BCC and PDAC T cell subcluster to determine their level of inter-cellular communication (Figure 2K, Methods). Signaling strength was inferred using the CellChat package by considering multiple measures of network centrality for each cluster, utilizing a manually-curated database of hundreds of ligand-receptor interactions (Jin et al., 2021). We find that nearly all BCC T cell subclusters are more dominant “senders” and “receivers” than their PDAC counterparts. In particular, due to the small size of the CD8^+^ memory T cell population in PDAC, it exhibits negligible inter-cellular communication, whereas CD8^+^ memory T cells in PDAC are extremely active. Additionally, proliferating T cells in BCC are the dominant senders and receivers, despite constituting 4% of the BCC T cell population; no equivalent subcluster was identified in the PDAC samples. Altogether, these results demonstrate that CD8^+^ T cells in BCC are substantially more active than their counterparts, both in aggregate and in the MHC and PD-1 pathways.

### 2.3 Differential Expression of MHC-I in Malignant Cells Is Associated With Response to PD-1 Therapy

**FIGURE 3:**
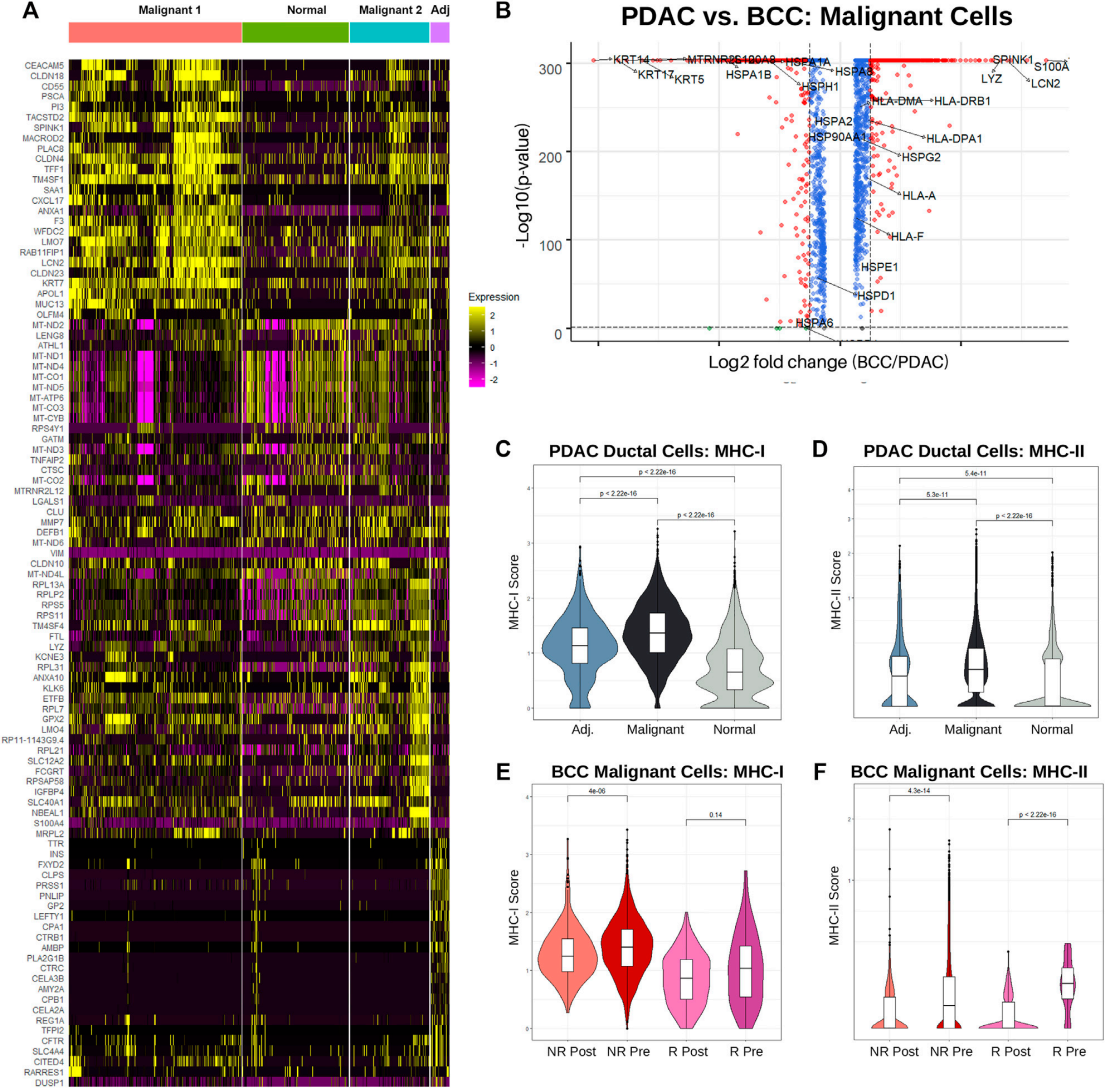
Increased MHC-I expression is associated with nonresponse to PD-1 therapy in BCC and PDAC. **(A)** Single-cell-resolution heatmap of top 20 most differentially expressed genes in each PDAC ductal cell subcluster. **(B)** Differential gene expression between BCC and PDAC malignant cells; higher gene expression in PDAC is denoted by a positive log2 fold change. **(C)** Violin plot of MHC-I score by cell in ductal cells. **(D)** Differential gene expression in malignant cells, PDAC vs BCC. **(E, F)** Violin plot of the **(E)** MHC-I and **(F)** MHC-II scores per cell in BCC malignant cells, classified by response (R/NR) and treatment (Pre/Post).

Multiple distinct subtypes of PDAC have been defined on the basis of significant inter-tumoral and intra-tumoral heterogeneity to develop personalized treatment strategies (Moffitt et al., 2015). To determine whether the two distinct malignant ductal cell subpopulations in our clustering (see Figure 1E) represent unique subtypes, we compared the marker genes for each PDAC ductal cell subcluster (Figure 3A). We find that the majority of the top markers for normal ductal cells in cancerous patients are mitochondrial genes (*MT-ND2*, *MT-ND1*, *MT-ND4*, *MT-CO1*, *MT-ND5*, *MT-ATP6*, *MT-CO3*, *MT-CYB*, *MT-ND3*, *MT-CO2*, *MTRNR2L12*, *MT-ND6*, and *MT-ND4L*), supporting previous research that mitochondrial metabolic reprogramming may be crucial to the progression of pancreatic cancers (Reyes-Castellanos et al., 2020). The Malignant 2 cluster was characterized by upregulation of several ribosomal protein (RP) genes, corroborating hypotheses that unique RP transcript expression can be utilized in defining unique cancer subtypes (Dolezal et al., 2018). Interestingly, ductal cells from adjacent samples exhibited elevated expression of marker genes for the Malignant 1 subcluster in comparison to ductal cells from cancerous samples. Although each malignant cell subcluster is dominated by a subset of the cancerous samples (Supplementary Figure S3A), suggesting that inter-tumoral heterogeneity led to the presence of two distinct PDAC subtypes in the dataset, we fail to identify concrete evidence linking either malignant cluster to the cancer subtypes defined in (Moffitt et al., 2015) and (Bailey et al., 2016).

While numerous studies have been conducted on the genetic markers of BCC and PDAC individually (Pellegrini et al., 2017) (Liu et al., 2020) (Tang et al., 2018) (Kunovsky et al., 2018), we present here the first direct comparison between the expression patterns of malignant cells in the two cancers (Figure 3B). We find that unsurprisingly keratins (namely *KRT5*, *KRT14*, and *KRT17*) which are marker genes for keratinocytes are overexpressed in BCC compared to PDAC by approximately an order of magnitude. Furthermore, many *HSP* genes are unexpectedly upregulated in BCC malignant cells, despite their well-known association with carcinogenesis and metastasis (Ciocca and Calderwood, 2005) (Wu et al., 2017). Expression of *HLA* genes (MHC-I and MHC-II) are slightly upregulated in PDAC malignant cells. The most upregulated genes in PDAC include *SPINK1*, known for contributing towards increased tumor proliferation and poor cancer prognosis (Mehner and Radisky, 2019); *TFF1*, which facilitates PDAC metastasis (Arumugam et al., 2011); and *S100A6*, a key diagnostic marker for PDAC (Leclerc and Vetter, 2015).

As the role of MHC-I and MHC-II in both BCC and PDAC tumors are well-established, we sought to compare the distribution of *HLA* gene expression between the two cancers. Using the same calculation for the MHC-I and MHC-II scores as Figures 2F–H, we find that on a cellular level, MHC-I expression is significantly upregulated in PDAC malignant cells compared to normal ductal cells from both cancerous and adjacent samples (Figure 3C). Interestingly, ductal cells from adjacent samples also had significantly higher MHC-I expression than those from normal samples. This provides more evidence that adjacent samples are not true negative controls. MHC-II expression followed the same trends, notably with a majority of non-cancerous ductal cells having zero expression (Figure 3D).

Looking at average MHC-I and MHC-II expression per patient between malignant and normal ductal cells, similar trends emerge. Whereas MHC-I expression is significantly elevated in malignant cells, no significant difference exists between average MHC-II scores, with the distribution of scores for normal ductal cells actually possessing a higher median and much greater variance (Supplementary Figure S3C). This suggests that there exists significant inter-tumoral variability in MHC-II expression, with the larger tumor samples having lower expression and therefore disproportionately shifting the cellular distribution downwards.

It appears that PDAC is non-responsive to treatment despite having already-elevated levels of MHC-I expression. To determine the relationship between MHC-I expression and response, we turned our attention to analyzing differences in MHC expression between BCC responders and nonresponders, both before and after treatment. Surprisingly, we find that regardless of response status, MHC-I expression slightly decreased post-treatment; however, nonresponders overall had higher expression (Figure 3E). This is likely due to a combination of greater initial tumor malignancy in non-responders and T cell exhaustion over time. Meanwhile, responders had significantly higher MHC-II expression pre-treatment, but both responders and non-responders experienced drastic reductions in expression post-treatment (Figure 3F). Whereas PDAC malignant cells exhibited greater similarities in MHC-I expression to BCC non-responders, MHC-II expression was more similar to BCC responders.

### 2.4 Machine Learning Reveals Divergent Immune Mechanisms in Response to PD-1 Blockade

With stark differences in immunogenicity, TMB, and tumor progression between BCC and PDAC, it is hardly surprising that the immune mechanisms implicated through PD-1 blockade in the two cancers are completely divergent. However, there exists greater similarity in the immunosuppressivity and immunogenicity between PDAC and melanoma, which exhibits a relatively high response rate to PD-1 blockade of 30–45% (Sun et al., 2020) (Ribas and Wolchok, 2018). To test whether the immune response of PDAC is more similar to BCC or melanoma, and whether differential gene expression can recapitulate these differences, we turn to machine learning. CD8^+^ cytotoxic T cells are most directly responsible for killing tumor cells and constitute the largest cluster in our datasets. Therefore, in this section we attempt to construct a supervised learning algorithm to predict whether individual CD8^+^ T cells originate from a patient responsive or nonresponsive to treatment. Patients with a high percentage of CD8^+^ T cells predicted to be responsive will therefore have a higher likelihood of response to PD-1 blockade.

Separate supervised learning algorithms were trained on both BCC and melanoma CD8^+^ pretreatment T cells, subsetted from (Yost et al., 2019) and (Sade-Feldman et al., 2018) respectively. The BCC dataset consists of 4,311 cells (229 effector, 1,104 exhausted, and 2,978 memory) from five responders and 1,571 cells (73 effector, 2,439 exhausted, 4,156 memory) from six nonresponders; the melanoma dataset consists of 1,512 cells from 17 responsive samples and 1,239 cells from 31 nonresponsive (32 total patients). The cells from (Sade-Feldman et al., 2018) were FACS sorted on CD45 ^+^ before plating and sequencing. Identification of CD8^+^ T cells in the melanoma dataset were taken directly from (Sade-Feldman et al., 2018) and (Dollinger et al., 2020).

**FIGURE 4:**
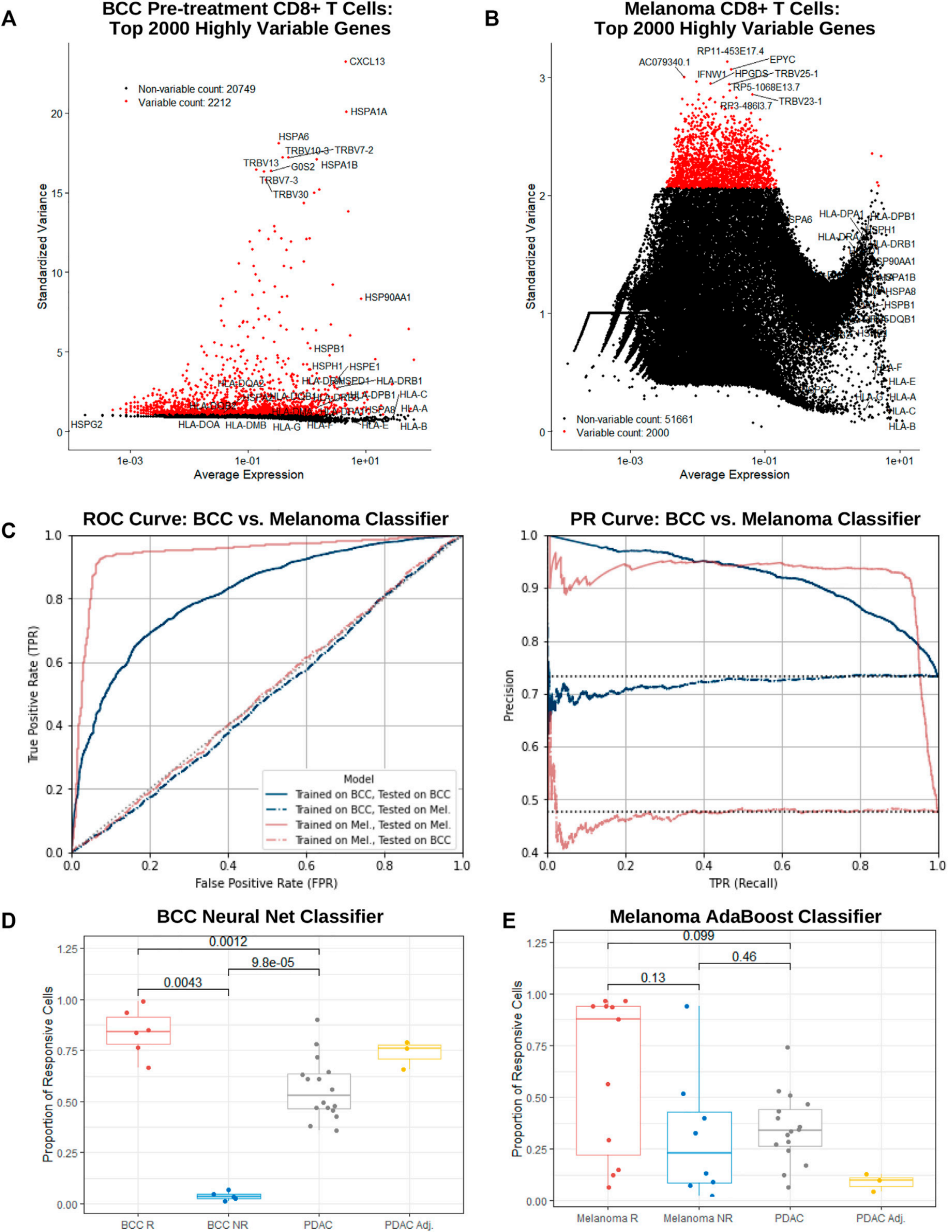
BCC, PDAC, and melanoma exhibit different immune mechanisms in response to PD-1 blockade. **(A,B)** Dot plot of average gene expression vs standardized variance of all CD8^+^ T cells in **(A)** BCC pre-treatment and **(B)** melanoma samples. **(C)** ROC and PR curve of a neural net (MLP) and AdaBoost classifier trained on the top 2000 highly variable genes in BCC and melanoma CD8^+^ T cells respectively; both models were subsequently tested on each dataset separately. **(D, E)** Proportion of CD8^+^ T cells classified as responsive to PD-1 blockade per patient by the **(D)** BCC neural net and **(E)** melanoma AdaBoost classifier.

Each dataset was first filtered to include only genes with expression detected in all three datasets (BCC, melanoma, and PDAC). Classifiers were then constructed on the BCC and melanoma CD8^+^ T cells through the sci-kit learn pipeline (Pedregosa et al., 2011), using only the top 2,000 highly variable genes in each dataset respectively (Figures 4A, B, Methods). Through benchmarking multiple classifiers against one another, we are able to identify the classification algorithm which most accurately responds to the features present in our datasets. With the exception of Naive Bayes, all classifiers demonstrated high training accuracy (>73%) on the BCC dataset; the best model was the multilayer perceptron (MLP) neural network, which achieved 96.7% testing accuracy on the original dataset after parameter optimization. Classifiers trained on the melanoma dataset were noticeably weaker, with training accuracy between 50 and 62%; after optimization, the best model was the AdaBoost, which achieved 60.7% testing accuracy. This could stem from the extremely high intratumor and intertumor heterogeneity observed in melanoma, which lowers predictive power (Grzywa et al., 2017). In addition, many of the melanoma patients were previously treated with other chemotherapeutics, potentially altering the immune environment and confounding the classification of responders.

To guard against overfitting, classifiers utilizing a lower number of most highly variable genes were constructed for both BCC and melanoma. Remarkably, in both datasets, predictive power remained notably strong until using just 20 or less genes. In particular, the 20-gene BCC classifier with 81% accuracy utilized *CXCL13*, *HSPA1A*, *HSPA6*, *HSPA1B*, *G0S2*, *XCL1*, *CCL4*, *FOS*, *GNLY*, *TRBV11-2*, *XCL2*, *KRT86*, *NMB*, *DNAJB1*, *CCL4L2*, *SOX4*, *ID3*, *HSP90AA1*, *NR4A1*, and *MT1G* (Supplementary Figure S4A, B). This suggests that traditional gene expression tests may be used as a quick predictor of response to PD-1 blockade with reasonably accuracy (60–80%) in BCC and melanoma.

Melanoma and BCC are known to exhibit very different immune mechanisms: whereas melanoma is immunogenic and demonstrates resistance to immunotherapy, BCC is relatively non-immunogenic and suffers from low immune cell recruitment and activation (Dollinger et al., 2020). To confirm these differences on a transcriptomic level, we tested the BCC classifier on the melanoma dataset and vice versa. As expected, both classifiers performed similarly to random chance (AUC = 0.501 and 0.486 respectively), providing support for the different immune evasive and suppressive mechanisms of the cancers’ response to PD-1 blockade (Figure 4C).

On a per-patient resolution, the vast majority of BCC CD8^+^ T cells from responders or nonresponders are classified as responsive or nonresponsive by the BCC neural net respectively (Figure 4D) – there exists a significant difference in the proportion of cells that are responsive between responders and nonresponders (*p* = 0.004 35). However, when applied to PDAC cells, slightly over half of the cells were declared responsive. Meanwhile, surprisingly no significant distinction (*p* = 0.13) can be made in comparing the percentage of cells classified as responsive between melanoma responders and nonresponders (Figure 4E), with several responders having only a small fraction of cells being classified as such. This indicates that construction of the classifier was likely biased towards samples with larger numbers of CD8^+^ T cells. The melanoma classifier furthermore identifies a mean of 33% of cells in a PDAC patient as nonresponsive, similar to melanoma nonresponders (*p* = 0.46) and significantly lower than responders (*p* = 0.099), although there exists significantly inter-patient variability. Under the assumption that the vast majority of PDAC patients would not respond to PD-1 blockade, it is evident that the melanoma classifier performs markedly better on the PDAC dataset than the BCC classifier. This suggests that similarities between resistance mechanisms between melanoma and PDAC may extend to CD8^+^ T cells in addition to macrophages (Zhu et al., 2014).

## 3 Discussion

To date, multiple studies of BCC have established its relative ease in prognosis and treatment; meanwhile, PDAC continues to evade early-stage detection and exhibits uniformly poor response to existing checkpoint immunotherapies. Consistent with existing literature, our direct comparison of the BCC and PDAC TMEs reveal that PDAC tumors foster a more immunosuppressive microenvironment compared to BCC (Foucher et al., 2018). In particular, although BCC is known to downregulate MHC-I expression (Dhatchinamoorthy et al., 2021), we find that PDAC suppresses both MHC-I and MHC-II expression in CD8^+^ T cells even more severely by a factor of 2–5. This further reinforces prevailing beliefs that BCC and PDAC utilize divergent immune mechanisms in combating tumor progression. However, through our novel identification of malignant ductal cells in the PDAC TME, we find that the two cancers exhibit similar expression of MHC genes, although MHC-I and MHC-II expressions are slightly elevated in PDAC.

Strikingly, we were able to construct a classifier to predict response to PD-1 blockade in BCC CD8^+^ T cells with near-perfect accuracy (97%). Even when considering data from only a handful of highly variable genes, responders and nonresponders were clearly distinguished. These results may suffer from overfitting due to the lack of suitable testing data: it is unknown whether the accuracy is artificially high due to the relative homogeneity of the TMEs of the 11 patients studied, or that the classifier will remain as successful in predicting the outcomes of other BCC cases. It is very likely that these models neglected to encompass the full spectrum of BCC subtypes, particularly nodular types with a high rate of heterogeneous morphological features both intra- and inter-tumorally (Pirie et al., 2018). However, our results strongly support that rapid and affordable testing of BCC patients, focusing on a small number (10–20) of genes, can accurately predict their response to checkpoint immunotherapies. Unfortunately, such clean results were not attained when training classifiers on the melanoma dataset or when testing either on PDAC; in these cases, our classifiers did not perform significantly better than random chance, mirroring previous efforts in the field (Banchereau et al., 2021).

The dearth of clinical data on the positive response of PDAC patients to PD-1 blockade leaves open multiple avenues of exploration. While the present study offers convincing evidence that PDAC, BCC, and melanoma lie in unique positions on the spectrum of immunogenicity, little is known of the exact changes in the immune landscape triggered by PD-1 blockade treatment, even in responders. A recent study on the same BCC dataset focusing on cell-cell communications offers several possible avenues of investigation, including the role of multiple tumor necrosis factor (*TNF*) pathways and a unique subtype of CD8^+^ T cells characterized by high expression of suppressive, cytotoxic, and heat shock protein genes (Jiang et al., 2021). Furthermore, there is a crucial need for further research into actionable mechanisms to overcome resistance to immune checkpoint inhibitors – current studies point to the potentiality of combination therapies in delivering individualized, multi-faceted remodeling of the TME (Ott et al., 2017) (Drake, 2012). Other hypothesized factors for immune resistance include tumor exploitation of the PD-1/PD-L axis, immuno-editing in tumor cells, the immunosuppressive effects of long non-coding RNAs (lncRNAs), and insufficient re-invigoration of exhausted CD8^+^ T cells (Drake et al., 2006) (Sun et al., 2020).

We chose to focus on the role of MHC-I and MHC-II expression in this investigation due to its well-established role in stimulating immune responses, as well as the obvious choice of PD-1/PD-L. However, various other genes and pathways associated with resistance to PD-1 blockade, such as *LAG-3* and the *IDO* pathway, were not studied in-depth (LaFleur et al., 2018) (Chocarro de Erauso et al., 2020). Additionally, recent studies have suggested that M2 macrophages and memory B cells play vital roles in directly affecting cancer cells (Dollinger et al., 2020) (Drake et al., 2006). With only six responders and five nonresponders in our BCC dataset, it is also likely that many cancer subtypes and diverse response mechanisms were not detected.

Despite the purely computational nature of the present study, our novel attempt to carry out quantitative comparisons of completely different cancers using scRNA-seq will facilitate a greater understanding of the immune landscape through the identification of both differences and similarities across different TMEs. Fundamentally, studying the activities of the same celltype in different TMEs serves an equivalent purpose to investigating the role of the same genes in different cancers. Although BCC and PDAC reside on opposite extremes of the spectrum of immunogenicity, the parallels that can be drawn between them will point the way towards establishing new immuno-oncology paradigms for more personalized and sophisticated immunotherapies.

## 4 Materials and Methods

### 4.1 Clustering

All clustering analyses were performed using Seurat (version 3.6) (Stuart et al., 2019). The UMI matrices for the BCC dataset (Yost et al., 2019) and PDAC dataset (Steele et al., 2020) were downloaded from GEO accession GSE123813 and GSE155698 respectively; the count matrix for the melanoma dataset (Sade-Feldman et al., 2018) was personally contributed by the authors of (Dollinger et al., 2020). No clinical trials were performed in the data acquisition or any other part of the preparation of this paper. The following procedures were applied to both the BCC and PDAC dataset; preparation and analysis of the melanoma dataset is separately dealt with in Section 4.4.

To exclude low-quality cells and empty droplets, we excluded all cells with less than 200 features detected; furthermore, we excluded all features that were not present in at least three cells. In preparation for clustering, we then followed the preprocessing steps detailed in (Stuart et al., 2019). Briefly, we first normalized the feature expression using the Seurat LogNormalization method with defaults, then applied linear transformation to shift the mean expression of each gene to 0 and the variance to 1. We then identified the top 2000 highly variable genes through calculating the “standardized variance” of each feature, which captures single-cell dispersion in the context of mean expression. Using these features, linear dimensional reduction was conducted on the normalized through PCA. The first 50 PCs for the BCC dataset and first 20 PCs for the PDAC dataset were used to construct a KNN graph; the number of PCs used was determined using the Seurat ElbowPlot function by identifying the cutoff at which the percentage of variance explained by each additional PC dropped significantly. The Louvian algorithm was then applied on the KNN graph to group cells together. The “granularity” of the clustering was determined by a resolution parameter which was set to 0.4 for BCC and 0.5 for PDAC (Seurat default = 0.3, higher resolutions correspond with a greater number of clusters).

To identify clusters, differential expression was performed to identify the top upregulated features in each cluster in comparison to all other clusters. Features were considered to be upregulated if at least 25% of cells in the cluster expressed the gene, the mean expression was greater by at least a factor of 2^0.25^, and the *p*-value was less than 0.05. The top 25 DEGs were then recorded and entered into Enrichr for gene enrichment analysis (Xie et al., 2021). Using a combination of different gene datasets (e.g. Human Gene Atlas and Mouse Gene Atlas) and common celltype marker genes, clusters were holistically identified. To ensure that equally-named clusters between BCC and PDAC (e.g. CD4^+^ T cells) were identified similarly and were suitable for downstream comparison, cluster identification was checked using marker genes identified in both (Yost et al., 2019) and (Steele et al., 2020).

To validate the results of our clustering of the BCC dataset, we constructed a heatmap to compare our cell labels with those provided in the metadata of GEO accession GSE123813 (Supplementary Figure S2A). Specifically, we calculated the percentage of cells in each metadata cluster that was classified into each self-identified cluster. As no celltype identification was supplied in the PDAC dataset, we were unable to do the same for this dataset.

### 4.2 Statistical Analyses of Datasets

All statistical analyses were performed in RStudio version 1.4. We merged the BCC and PDAC datasets together without batch correction to compare expression of key marker genes in each cluster (Figure 1F) and relative population sizes of each cluster (Figure 1G); integration was not necessary as no new clustering was conducted. To calculate the breakdown of each cluster in the merged BCC + PDAC dataset between BCC responders, BCC nonresponders, PDAC cancerous samples, and PDAC adjacent samples (Figure 1G), we first normalized the total size of each of the four batches so that each batch would appear to have the same total number of cells. We then determined the normalized proportion of cells in each cluster that belonged to each batch by dividing the normalized population size of each batch in each cluster by the total normalized population of the cluster.

To compare the proportion of all cells that were identified as T cells (Figure 2B), we first determined the number of cells in each patient sample. Then, we calculated the fraction of these cells that were labeled as either CD4^+^, CD8^+^ effector, CD8^+^ memory, CD8^+^ exhausted, regulatory (Tregs), proliferating, NK, or miscellaneous T cells for each sample. To determine whether differences in this percentage between different groups were statistically significant, we performed Wilcoxon tests using the stat_compare_means function in the ggpubr package, version 0.4.0. A *p*-value lower than 0.05 was considered to be statistically significant. The same procedure was repeated for Figure 2C, D.

Comparisons of the distribution of expression of any particular gene between different clusters and/or categories (Figures 2F–I) were conducted by first taking the data from the normalized UMI matrix, then exponentiating all of the values so that any comparisons occur in non-log space. The distribution of these values were plotted using the ggviolin function in the ggpubr package; statistical significance was determined through a Wilcoxon test. Identical procedures were used in Figures 3C–F. The same method was also used to determine the *p*-values in Figure 2J; however, the log2-fold difference was calculated by dividing the mean expression of the particular gene of all BCC cells to the mean expression of all PDAC cells in the particular cluster. Mean expression was calculated using the AverageExpression function in Seurat and was therefore performed on raw data counts, as opposed to scaled/normalized data.

To perform full differential gene expression between two clusters (Figure 1E, Figure 2E, and Figure 3B), the Seurat objects of the clusters of interest were first merged together. Then, the EnhancedVolcano function from the Bioconductor package was used to generate the volcano plots in Figure 1E and Figure 3B. Features that were considered to be differentially expressed were those with a *p*-value <0.05 and a log2-fold absolute change greater than 0.5. For Figure 2E, the log2-fold change for every gene was ordered and normalized (*μ* = 0, *σ* = 1); then, the 2000 genes with the lowest absolute value normalized log2-fold change were identified as the genes with most similar expression.

Heatmaps of gene expression were generated using the DoHeatmap function from Seurat. The genes displayed are the top *n* DEGs per cluster (*n* = 3 and 25 for Figure 1F and Figure 3A respectively).

### 4.3 Inference of Intercellular Communication Network Strengths

Cell-cell communication was determined using CellChat version 1.1 (Jin et al., 2021). Briefly, the cell-cell communication network was inferred by calculating the interaction probabilities, which is directly dependent on average gene expression, for each ligand-receptor pair in the CellChat database. The sum of communication probabilities of outgoing signaling from and incoming signaling to a particular cluster determines its outgoing and incoming interaction strength respectively, as plotted in Figure 2K.

### 4.4 Supervised Learning: Prediction of Response to PD-1 Blockade in BCC and Melanoma

Classifiers were constructed on CD8^+^ T cells in BCC and melanoma (Figure 4). The BCC dataset consisted of all pre-treatment cells identified as CD8^+^ effector, CD8^+^ memory, or CD8^+^ exhausted. The melanoma dataset consisted of all CD8^+^ T cells as identified in (Dollinger et al., 2020).

All machine learning was conducted in Python using the scikit-learn package (Pedregosa et al., 2011). Identification of the top 2000 highly variable genes (Figures 4A, B) recapitulated the process described in Section 4.1. To determine the best model in differentiating cells from responders and nonresponders, nine separate classifiers were trained separately on the BCC and melanoma datasets:

• Nearest Neighbors (sklearn.neighbors.KNeighborsClassifier): three neighbors
• Linear SVM (sklearn.svm.SVC): linear kernal, *C* = 0.025
• RBF SVM (sklearn.svm.SVC): *γ* = 2, *C* = 1
• Gaussian Process (sklearn.Gaussian_process.GaussianProcessClassifier)
• Decision Tree (sklearn.tree.DecisionTreeClassifier): max_depth = 5
• Random Forest (sklearn.ensemble.RandomForestClassifier): max_depth = 5, max_estimators = 10, max_features = 1
• Neural Net (sklearn.neural_network.MLPClassifier): *α* = 1, max_iterations = 1000
• AdaBoost (sklearn.ensemble.AdaBoostClassifier)
• Naive Bayes (sklearn.naive_bayes.GaussianNB)
• Quadratic Classifier (sklearn.discriminant_analysis. QuadraticDiscriminantAnalysis)

All parameters used are the default ones unless listed explicitly. Models were trained on 80% of the dataset and tested on the remaining 20%. During parameter optimization, the mean score from five-fold cross validation was used to evaluate accuracy. The best classifier was chosen as the one with the highest accuracy, i.e. proportion of true positives and true negatives. For BCC CD8^+^ T cells, this was the neural net classifier; for melanoma CD8^+^ T cells, this was the AdaBoost classifier. After parameter optimization, the BCC classifier had an architecture of one hidden layer with 20 nodes, a rectified linear unit (relu) activation function (*f*(*x*) = max(0, *x*)), and a stochastic-gradient based optimizer (adam); the learning rate is *α* = 1 and all other hyperparameters are equal to function defaults. The melanoma classifier has an architecture of 500 estimators using the SAMME. R real boosting algorithm and a learning rate of *α* = 1. To ensure consistency, all classifiers trained using a reduced number of highly variable features (Supplementary Figure S4A, B) used the same architectures.

To calculate the proportion of cells in each patient that are classified as responsive (Figures 4D, E), the BCC neural net classifier was tested on the BCC pretreatment and PDAC datasets, and the melanoma AdaBoost classifier was tested on the melanoma and PDAC datasets. The number of CD8^+^ T cells classified as responsive in each patient was then divided by the total number of CD8^+^ T cells in each patient. To determine whether the results were statistically significant, a Wilcoxon test was performed using the stat_compare_means function in ggpubr; *p*-values less than 0.05 were considered as significant.

## Data Availability

Only publicly available datasets were analyzed in this study. This data can be found here: GEO accession GSE123813 for BCC and GSE155698 for PDAC. The melanoma dataset was directly provided by the authors of Sade-Feldman et al. and is available upon request. All code generated in preparing this article is publicly available at the following GitHub repository: https://github.com/rliu7926/bcc-pdac-pd1-blockade.

## Supplementary Material

Supplementary Figure S1 Visual abstract. **(A)** BCC and PDAC dataasets are clustered and labeled through Seurat,resulting in the novel identification of malignant ductal cells in PDAC (Section 2.1). **(B)** Several analyseswere performed on T cells and malignant cells from both datasets, focusing on population subcluster sizes,differential gene expression, and cell-cell signaling (Section 2.2/Section 2.3). **(C)** Machine learning classification models were successfully utilized to predict whether individual CD8+ T cells in BCC and PDAC would respond to PD-1 blockade; however, these models are not transferrable onto PDAC (Section 2.4).

Supplementary Figure S2 Validation of clustering in BCC and PDAC datasets. **(A)** Heatmap depicting the proportion of cells in each cluster of the original paper belonging to each cluster defined in this paper. **(B, C)** Dot plot of the expression of **(B)** CD8+ T cell and **(C)** ductal cell marker genes in PDAC. Color denotes the average expression across all cells in a subcluster, scaled per gene; size denotes the percentage of cells with positive expression within the subcluster.

Supplementary Figure S3 MHC expression in malignant cells of BCC and PDAC. **(A, B)** Breakdown of **(A)** PDAC ductal cell and **(B)** BCC malignant cell clusters by patient. **(C, D)** Paired comparison of MHC-I and MHC-II scores per patient in **(C)** malignant vs. non-malignant PDAC ductal cells and **(D)** pre-treatment vs. post-treatment BCC malignant cells. Horizontal boxplots represent the log2-fold difference in the MHC score per patient between the two batches; the T-test calculates the likelihood that on average, there is no difference in the score.

Supplementary Figure S4 Supervised classification of CD8+ T cells in BCC and melanoma based on the top *n* highlyvariable genes. **(A, B)** ROC and PR curves for classifier (architecture described in Methods) trained on the top n highly variable genes in **(A)** BCC and **(B)** CD8+ T cells. **(C)** Comparison of the proportion of top *n* highly variable genes that are common between BCC and melanoma CD8+ T cells. **(D)** Fold change differential expression of MHC and HSP genes between BCC and melanoma CD8+ T cells; positive values indicate greater expression in BCC.
