# Supplementary material for: Machine Learning of Single Cell Transcriptomic Data From anti-PD-1 Responders and Non-responders Reveals Distinct Resistance Mechanisms in Skin Cancers and PDAC
Source: Front Genet. 2022 Feb 1;12:806457. doi: 10.3389/fgene.2021.806457 (PMC8844526; doi:10.3389/fgene.2021.806457)
Supplement: Supplementary file 1 [file DataSheet1.pdf]

Supplementary Material

1 SUPPLEMENTARY TABLES AND FIGURES

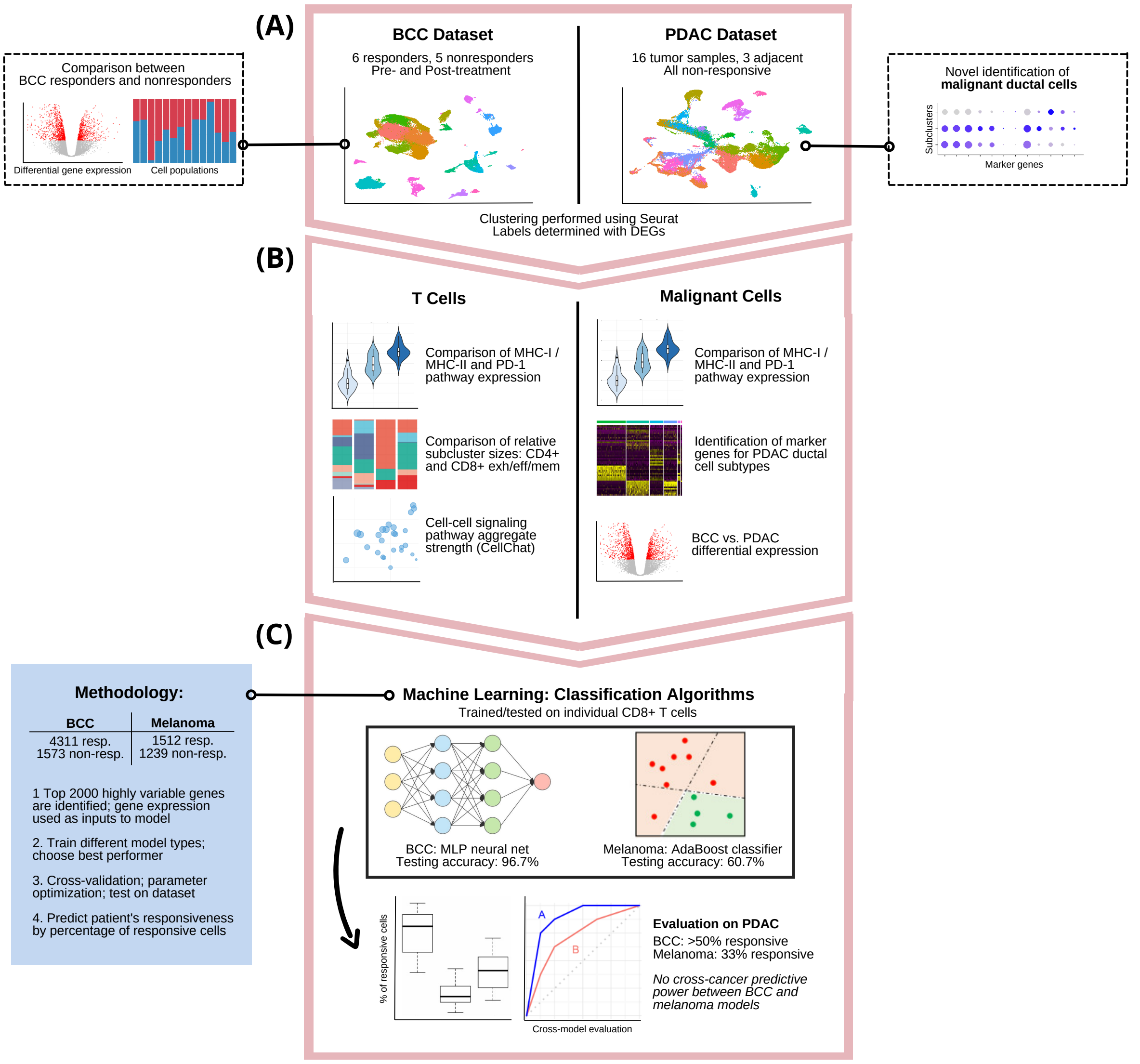

Figure S1. Visual abstract. **(A)** BCC and PDAC dataasets are clustered and labeled through Seurat, resulting in the novel identification of malignant ductal cells in PDAC (Section 2.1). **(B)** Several analyses were performed on T cells and malignant cells from both datasets, focusing on population subcluster sizes, differential gene expression, and cell-cell signaling (Section 2.2/2.3). **(C)** Machine learning classification models were successfully utilized to predict whether individual CD8+ T cells in BCC and PDAC would respond to PD-1 blockade; however, these models are not transferrable onto PDAC (Section 2.4).

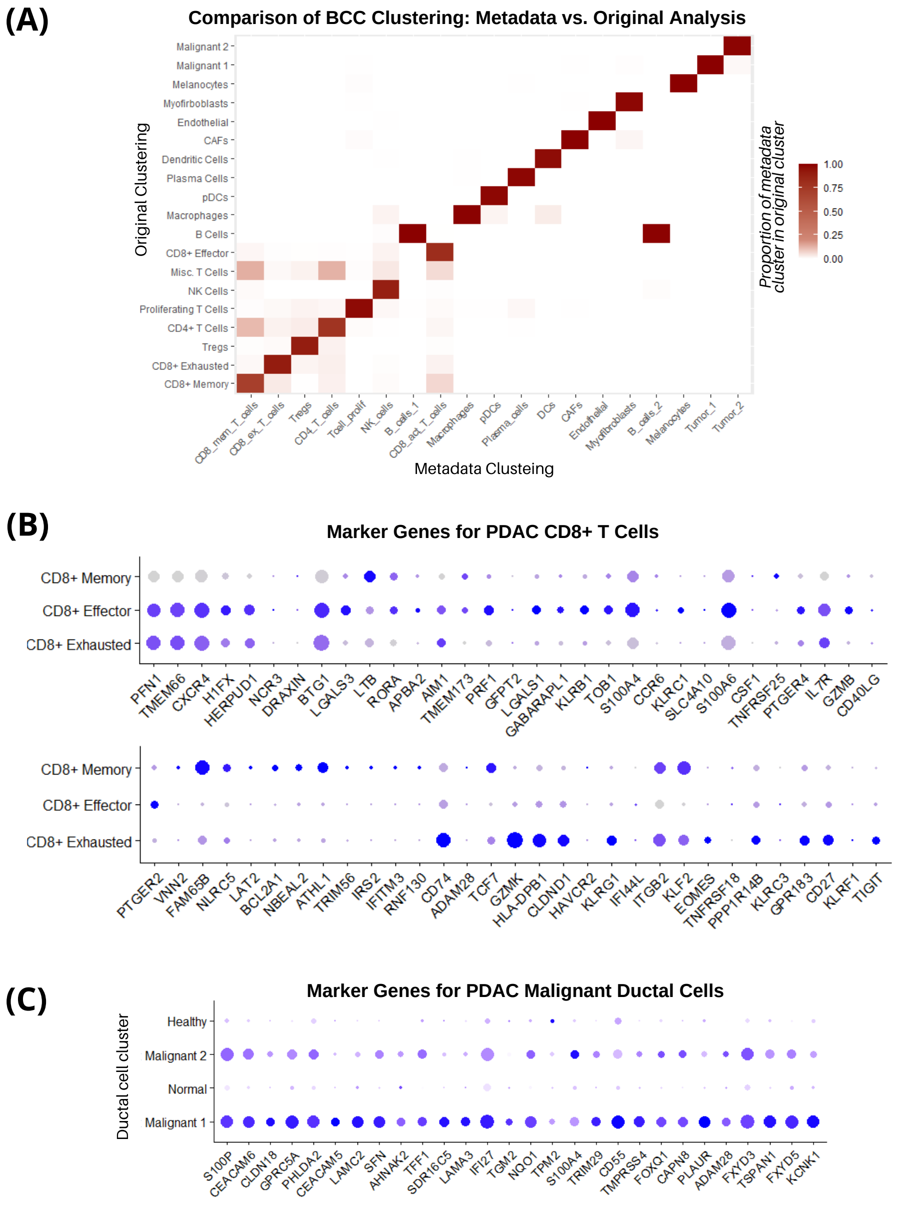

Figure S2. Validation of clustering in BCC and PDAC datasets. **(A)** Heatmap depicting the proportion of cells in each cluster of the original paper belonging to each cluster defined in this paper. **(B,C)** Dot plot of the expression of **(B)** CD8+ T cell and **(C)** ductal cell marker genes in PDAC. Color denotes the average expression across all cells in a subcluster, scaled per gene; size denotes the percentage of cells with positive expression within the subcluster.

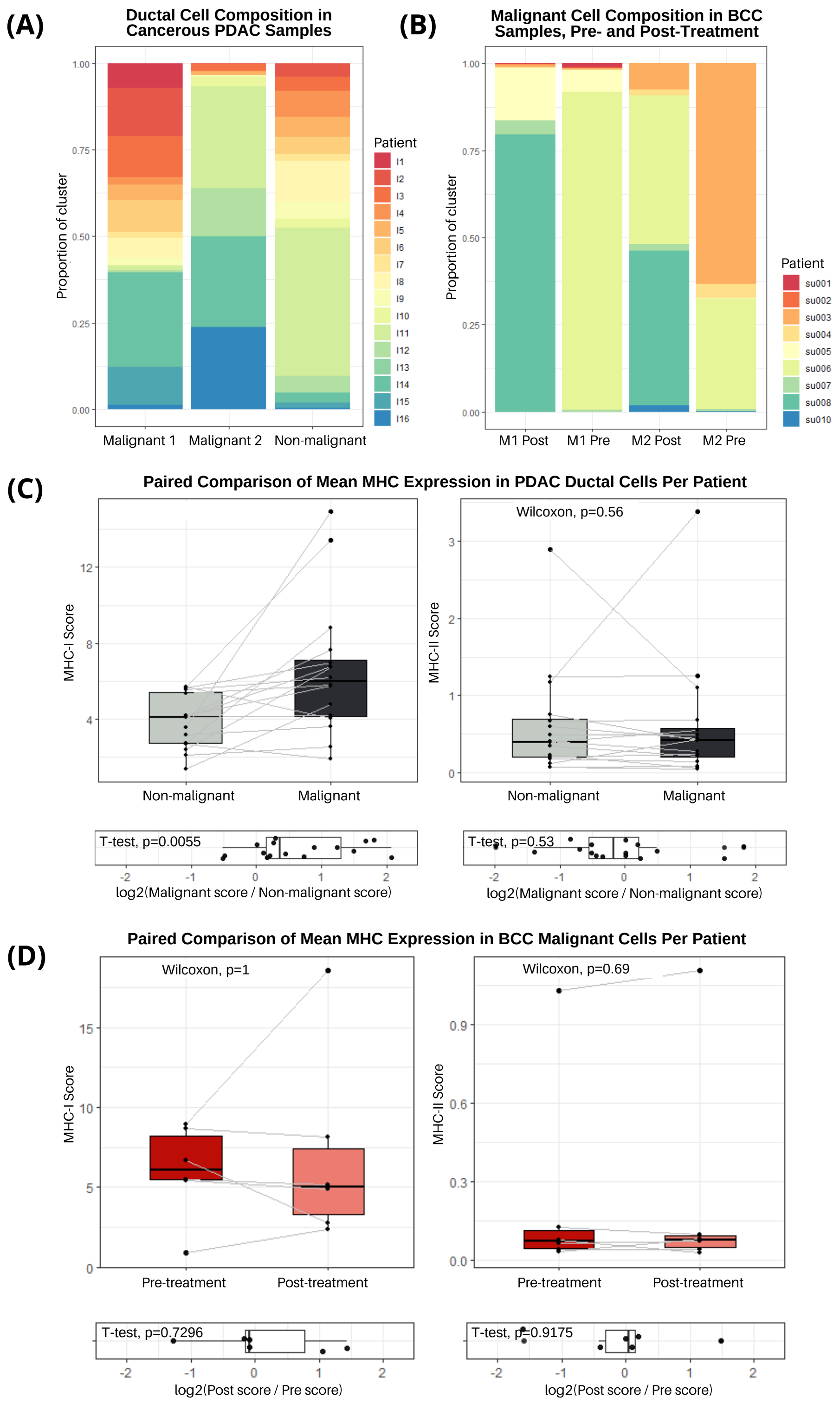

Figure S3. MHC expression in malignant cells of BCC and PDAC. **(A,B)** Breakdown of **(A)** PDAC ductal cell and **(B)** BCC malignant cell clusters by patient. **(C,D)** Paired comparison of MHC-I and MHC-II scores per patient in **(C)** malignant vs. non-malignant PDAC ductal cells and **(D)** pre-treatment vs. post-treatment BCC malignant cells. Horizontal boxplots represent the log2-fold difference in the MHC score per patient between the two batches; the T-test calculates the likelihood that on average, there is no difference in the score.

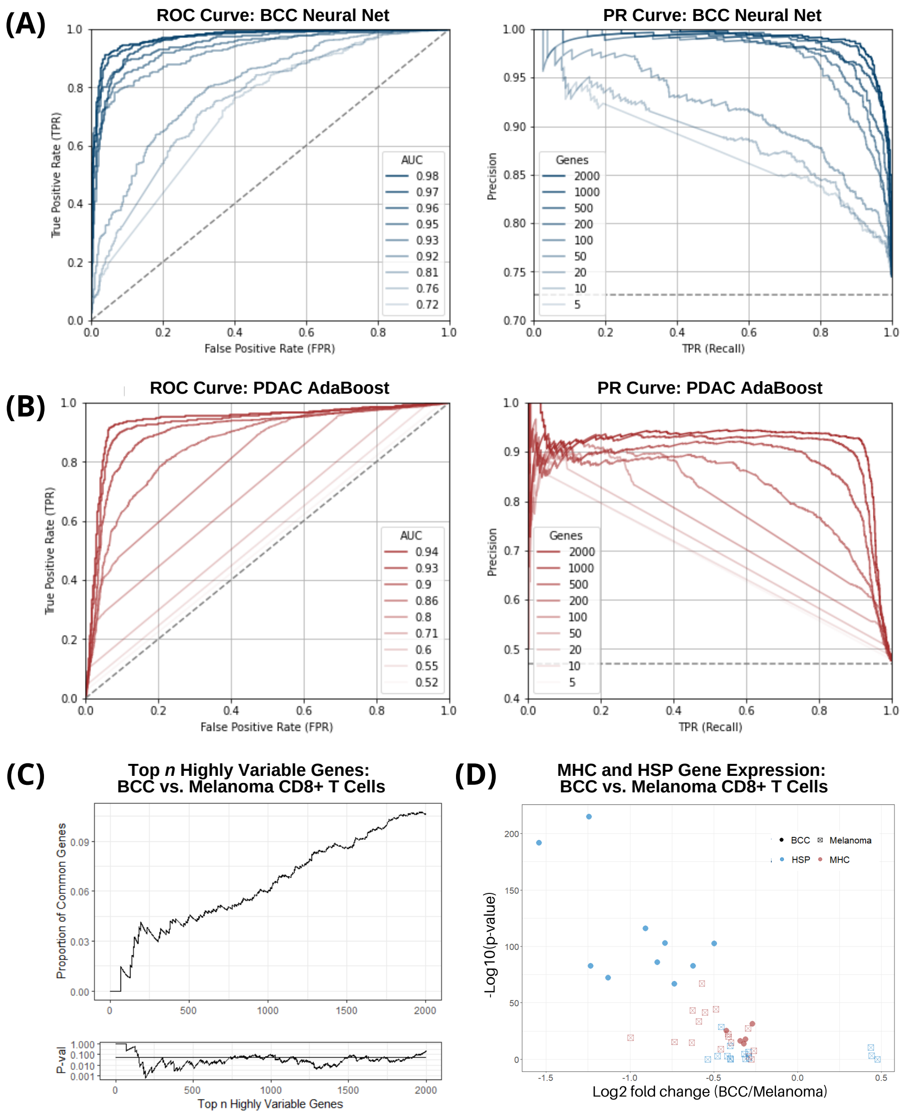

Figure S4. Supervised classification of CD8+ T cells in BCC and melanoma based on the top *n* highly variable genes. **(A,B)** ROC and PR curves for classifier (architecture described in Methods) trained on the top *n* highly variable genes in **(A)** BCC and **(B)** CD8+ T cells. **(C)** Comparison of the proportion of top *n* highly variable genes that are common between BCC and melanoma CD8+ T cells. **(D)** Fold change differential expression of MHC and HSP genes between BCC and melanoma CD8+ T cells; positive values indicate greater expression in BCC.
